# An Examination of the Four-Part Theory of the Chinese Self: The Differentiation and Relative Importance of the Different Types of Social-Oriented Self

**DOI:** 10.3389/fpsyg.2017.01106

**Published:** 2017-06-30

**Authors:** Chien-Ru Sun

**Affiliations:** Department of Psychology, National Chengchi UniversityTaipei, Taiwan

**Keywords:** the four-part theory, Chinese self, social-oriented self, individual-oriented self, other-oriented self, the familistic (group)-oriented self, relationship-oriented self

## Abstract

Because culture has a deep and far-reaching influence, individuals who grew up within different cultures tend to develop different basic self-constructions. With respect to the Chinese under the influence of Chinese culture, Yang proposed the concepts of individual-oriented self and social-oriented self. He argued that, besides the individual-oriented self, the social-oriented self of the Chinese contains three types of self: the relationship-oriented self, the familistic (group)-oriented self, and the other-oriented self. The theory proposed that the Chinese self is appropriately covered only through this four-part theory of the Chinese self. However, this remains to be tested; whether these three types of sub-level “selves” can be effectively triggered, along with their relative importance. This study examines the four-part theory of the Chinese self. Through photo priming, Experiment 1 shows that the three types of social-oriented self are differentiated from each other and can be individually triggered. In Experiment 2, the importance of the three types of self was investigated, adopting the concept of limited self-regulation resources to design scenarios. The participants were asked to make counterarguments about the notion of each of the three types of self, with performance in the subsequent task serving as the main dependent variable. In Experiment 3, the relative importance of the three types of self was examined by investigating the choices made by individuals within the context of conflict under the three orientations of the social-oriented self. Overall, results of the experiments showed that the Chinese have a four-part self with the importance of the other-oriented self as the most remarkable.

## Introduction

When investigating the differences in individuals' psychology or behavior and the interpersonal interaction process, most studies have made generalized comparisons and engaged in discussions from the cultural perspective (Triandis, 1995). Individuals influenced by an individualistic culture mostly focus on the realization of personal goals, the protection of personal interests, and the individual's independence and privacy, believing that individuals ought to seek self-sufficiency and avoid relying on others. Typical representatives of such cultures include those of some American and European countries such as the United States, Britain, and Canada. In contrast, the impact of a collectivist culture is very different: in such cultures, individuals emphasize their responsibilities to the group or others and believe that people need to support each other. Within a collective culture, helping either others or the group to achieve is even more important than self-actualization (Dion and Dion, 1993; Triandis, 1995). Examples of countries in which a collectivist culture prevails are Latin American and Asian countries such as Venezuela, China, and Peru.

Subsequent cross-cultural studies have also noted that the impact of culture is both deep and far-reaching and that individuals who developed in different cultures have long been molded into different shapes. For example, Markus and Kitayama (1991, 2010) argued that the Western concept of self is a concept of independent self that emphasizes the self's independence and uniqueness, believing that individuals must discover and demonstrate inherent personal qualities to distinguish from others. In contrast, individuals under the influence of Eastern cultures primarily aspire to an interdependent self that is both flexible and subject to change, stressing coordination between an individual and the social environment through appropriate personal behavior. Individuals with an interdependent self tend to seek a harmonious relationship with others and hope to help others achieve their goals.

Markus and Kitayama (1991, 2010) argued that these two different self-construals—i.e., two different basic self-schemas—would cause changes in how an individual assesses, organizes, and regulates his/her own experience and behavior. Furthermore, Singelis (1994) argued that although the “self” as conceived by Westerners is dominated by the *independent self*, while the “self” as conceived by Asians is dominated by the *interdependent self*, it is not necessarily true that the independent self and the interdependent self cannot coexist. Many subsequent studies have agreed with this view; For example, Kühnen and Oyserman (2002) demonstrated that they could effectively trigger different types of self in an individual and that when an individual's interdependent self was triggered, the individual tended to observe the surrounding context. However, when the individual's independent self was triggered, he/she was inclined to focus on the task itself.

## The chinese self

What is the self-construction of the Chinese under the influence of the Confucian culture? The connotations of the individual- and social-oriented self as proposed by Yang (1993, 1995) largely correspond to the concepts of independent self and interdependent self, respectively. Yang (1993) further defined the individual-oriented self as a combination of a tendency toward high personal autonomy and low homonymy that emphasizes an individual's personal achievement, performance, uniqueness and autonomy. At the meantime, Yang defined the social-oriented self as one that combines a tendency toward high homonymy (with the surrounding environment) and low personal autonomy, emphasizing that this type of self which attaches importance in order to maintain harmonious interpersonal relationships, accountability, and responsibility and requires appropriate personal behaviors such that individuals position themselves according to their relationship with others.

Yang (1993) also stressed that these two types of “self” can co-exist; in essence, within the Chinese culture, the importance of the social-oriented self cannot be ignored. This theory was supported later with the studies made by Lu et al. (2008) and Lu (2008), which they argued that the Chinese have a bicultural self, wherein that both the individual-oriented self and the social-oriented self are important. Later on, Yang et al. (2010) further attempted to understand and construct the bicultural self of the Chinese from the perspective of an individual's development stages and have gained some initial support for their notion.

In addition to analysis and research at the theoretical level, Sun and Wang's (2005) experiment found that among Chinese, the social-oriented self was the main source of positive self-evaluation among individuals because when the social-oriented self is threatened, the affirmation of important relations (e.g., with parents) that also belong to the relationship-oriented self can restore the balance of an individual's self, however, self-affirmation originating from the independence-oriented self seems irrelevant to restoring the balance of the social-oriented self. Sun (2004) also employed the research paradigm of “false uniqueness bias” and the “self-handicapping paradigm” and demonstrated that the Chinese did not show obvious modesty or self-effacing tendencies, and the tendency toward self-enhancement demonstrated in the social-oriented self was more remarkable than that demonstrated in the individual-oriented self. Lastly, Kurman (2001) showed that the Chinese manifest self-enhancement in their communal traits. The results of these studies largely show that among the Chinese, the social-oriented self is more important than the individual-oriented self.

## Yang's four-part theory of the chinese self

Does the distinction between the individual-oriented self and the social-oriented self suggest that complete understanding of the Chinese self has been achieved? The answer is no. In 2004, based on his previously proposed theory of the relation between the individual-oriented self-and the social-oriented self, Yang further established “the four-part theory of the Chinese self,” emphasizing that the social-oriented self contains three different types of self [relationship-oriented self, familistic (group)-oriented self, and other-oriented self], and therefore, the four-part theory can completely cover the Chinese self (Yang, 2004).

In previous studies on self, Western psychologists have regarded the subjective self (i.e., I-self) as an observer, an information handler or a cognition constructor while regarding the objective self (i.e., Me-self) as either the object to be observed/perceived or the object of cognition construction (e.g., Harter, 1999). This notion stresses that the I-self possesses perception, cognition, and evaluation functions, whereas the Me-self is only the object or target that receives these functions. On the one hand, Yang (2004) believes that this notion is narrow-minded; on the other hand, he emphasizes that “for people living in countries with starkly different historical, social, and cultural background, the principles, the ways and the contents adopted by the I-self when assuming the functions or playing the roles can be vastly different, so the resultant Me-self can also be very different” (p. 21). Yang further stresses that Western society has primarily been influenced by Christian culture, whereas Chinese society has been deeply influenced by Confucian culture. The two societies have drastically different historical, social and cultural aspects, resulting in significant differences between Westerners and Chinese with respect to the I-self and the Me- self.

Yang (2004) believes that within the context of Chinese culture, “the relationship between two persons under the equality context, the relationship between two persons under authoritarian context, the communal context of family and the generalized context of others are the most core interactive modalities in the daily life of Chinese people and make up the main parts of life in the Chinese society. The characteristics, connotations and operation principles are different for the aforementioned four modalities, and the modalities have their respective interactive ways and develop into four corresponding orientations over time, i.e., relationship orientation, authoritarian orientation, familistic (group) orientation and “other” orientation” (p. 22). Yang also believes that in terms of an individual's self-operation, Chinese people's four major social life modalities can be regarded as major interaction modalities in which the I-self assumes various functions and implements various acts, whereas individuals living in the modalities blend in from young to old and are naturally capable of skillfully and efficiently operating in appropriate social interaction modes after undergoing the processes of socialization, differentiation, and even automation.

Therefore, Yang (2004) stresses that for Chinese people, “in the process of self-development, the individual's performance and interaction effectiveness in the four modalities are the main objectives or object for the Chinese I-self to observe, inspect, perceive, think, reflect, judge, evaluate, plan, organize, control, manipulate, adjust, and correct, thereby giving rise to the four types of Me-self” (p. 21). For reasons of brevity, Yang combines the relationship orientation and authoritarian orientation into one that retains the name “relationship orientation.” In other words, the social-oriented self can be subdivided into three types of self—i.e., the relationship-oriented self, the familistic (group)-oriented self, and the other-oriented self—which together with the individual-oriented self comprise the four-part Chinese self. Yang argues that these four types of self represent Chinese people's methods of interacting with others in the four modalities, which are mutually distinctive. Yang further compares the similarities and differences among the four types of self with respect to the following 15 psychological aspects: (1) Dominant trend of adaptation; (2) Target of interaction; (3) Contextualization; (4) Role involvement; (5) Object of identification; (6) Type of identity; (7) Sense of responsibility; (8) Mode of self-consistency; (9) Primary motivation; (10) Essential affection; (11) Target of emotional attachment; (12) Type of self-actualization; (13) Type of self-concept; (14) Type of self-esteem; and (15) Type of happiness (Yang, 2004, see Table 1).

**Table 1:** Summary of a systematic conceptual comparison of individual- and social-oriented selves in terms of 15 major aspects.

		**Social-oriented Self**		
**Aspect**	**Individual-oriented self**	**Relationship-oriented self**	**Familistic(group)-oriented self**	**Other-oriented self**
1. Dominant trend of adaptation	Autonomous	Homonomous (union with another person)	Homonomous (union with one's family or some other membership group)	Homonomous (union with the non-specific others)
2. Target of interaction	One's own self or person	A related person	One's family or some other membership group	Non-specific others (the generalized other)
3. Contextualization	Decontextualization	Relationship-contextualized	Family (group)-contextualized	Non-specific-others-contextualized
4. Role involvement	Moderate social role involvement	High social role involvement in a relationship	High social role involvement in one's family or some other membership	Moderate social role involvement in the non-specific others
5. Object of identification	Identification with oneself (reflexive identification)	Identification with a related person (relational identification)	Identification with one's family or some other membership group (familial or group identification)	Diffuse social identification
6. Type of identity	Personal identity	Relational identity	Familial (group) identity	Diffuse social identity
7. Sense of responsibility	Personal responsibility	Reciprocal responsibility for the role partner in a specific relationship	Familistic responsibility for one's family or some other membership group	One-sided, self-imposed responsibility for the non-specific others
8. Mode of self-consistency	Self-consistency across situations and time (self-centered self-consistency)	Self-consistency with respect to specific dyadic relationships (relationship-specific self-consistency)	Self-consistency with respect to one's family or some other specific social group (group-specific self-consistency)	Self-consistency with respect to the non-specific others (other-oriented self-consistency)
9. Primary motivation	Agency needs (e.g., needs for self-reliance, autonomy, independence, self-acceptance, personal achievement, personal efficacy, personal superiority, individual-oriented self-esteem, and self-enhancement)	Relational needs (e.g., needs for dependence, interdependence, relational acceptance, relational sharing, relational face, relationship-oriented self-esteem, and self-enhancement)	Belongingness needs (e.g., needs for self extension, a larger self, group acceptance, group protection, group efficacy, group achievement, group glory, group face, familistic(group)-oriented sell-esteem, and self-enhancement)	Face-related needs (e.g., needs for public face, reputation, respect from the non-specific others, other-oriented self-esteem, and self-enhancement)
10. Essential affection	Narcissistic affects (e.g., self-love, self-respect, self-envy, self-glory, hedonic pleasure, personal happiness, feelings of individual-oriented self-esteem, and self-enhancement)	Role-specific dyadic affects (e.g., filial love, marital love, brotherly love, affects between teacher and pupil, feelings of gaining and losing relational face, feelings of relationship-oriented self-esteem and self-enhancement, feeling of relational shame)	Familistic (group)-oriented affects (e.g., familial love, group glory, feelings of belongingness, unity, and security, feelings of gaining and losing group face, feelings of familistic(group)-oriented self-esteem and self-enhancement, feeling of group shame)	Other-oriented affects (e.g., feelings of gaining and losing face, reputation, and respect from others, feelings of other-oriented self-esteem and self-enhancement, feeling of public shame)
11. Target of Emotional attachment	Attachment to one's self	Attachment to another person as the role partner in a specific relationship	Attachment to one's family or some other membership group	Attachment to the non-specific others
12. Kind of self-actualization	Individual-oriented self-actualization	Relationship-oriented self-actualization	Familistic (group)-oriented self-actualization	Other-oriented self-actualization
13. Kind of self-concept	Individual-oriented self-concept	Relationship-oriented self-concept	Familistic (group)-oriented self-concept	Other-oriented self-concept
14. Kind of self-esteem	Individual-oriented self-esteem	Relationship-oriented self-esteem	Familistic (group)-oriented self-esteem	Other-oriented self-esteem
15. Kind of well-being	Individual-oriented well-being	Relationship-oriented well-being	Familistic (group)-oriented well-being	Other-oriented well-being

*From Yang (2004)*.

Overall, Yang's so-called individual-oriented self refers to the demonstration and play of the characteristics and features that an individual uniquely possesses, whereas the relationship-oriented self emphasizes interaction relations in interpersonal modalities such as horizontal modality (e.g., husband and wife) and vertical modality (e.g., parents and children). The so-called familistic (group)-oriented self refers to the interaction relations of an individual with his or her clan and family both within and without the clan (or family), whose interaction history and connotation is based on Chinese familism. Yang also emphasizes that this type of familistic (group)-oriented interaction can be either subject to generalization or transferred to groups outside the family (e.g., work or business organizations); accordingly, family orientation also represents group orientation. Yang also proposed the so-called “other” orientation, referring to interaction relations with non-specific others under certain circumstances in which “non-specific others” are defined as a large number of anonymous “generalized others” with unknown faces. For example, within the sayings “too shameful to meet the clansmen” and “I wonder what others would think”, the “clansmen” and “others” are both “non-specific others” as defined by Yang. At such a moment, the individual self is related to these generalized others and is called the other-oriented self.

Yang (2004) also stresses both that the contexts under which different types of self form are different and that the individual's needs/motives vary. Consequently, the ultimate self-evaluation aspired by the individual will also be different. The individual-oriented self is decontextualized, attaches importance to autonomy and independence and looks forward to making achievements through the individual's ability and performance. The relationship-oriented self mainly uses relationships between two persons as the context, attaching importance to each person's roles and hoping to gain the other's recognition and acceptance. The familistic (group)-oriented self uses family (group) as the context in which the individual expects to play an appropriate role and be accepted by the family (group) to meet his or her identity-related needs. The other-oriented self cares about non-specific others and hopes that the individual's words and deeds can satisfy public expectations and be honored, earning a face before the public.

Indeed, this concept of the multi-part self is not unique to Yang: Greenwald and Breckler (1985), Sedikides and Brewer (2001), and Cross et al. (2003) have also proposed the idea of three types of self. The three types of self that they propose include the individual or private self, the relational self and the collective self. The individual or private self refers to characteristics unique to the individual, the relational self refers to the self-shared by the individual with important others and the collective self is the part of self that spans a group. Yang argues that these discussions of self are inadequate because distinctions of selves are defined only based on the number of people (one person, two or more persons) with which the self is involved: they neither are truly rooted in the local culture nor consider the characteristics of the culture pertaining to the self being discussed.

Yang's four-part theory of the Chinese self is founded on Chinese culture and supported by results of some preliminary studies; for example, Lu and Yang (2005) note that the achievement to which Chinese people aspire is rather different from that of the Westerners and should be divided into individual-oriented self-actualization and social-oriented self-actualization. In developing the scale of individual-oriented self-actualization and social-oriented self-actualization, Yang and Lu (2005) attempt to include items on different orientations of the self [including the relationship-oriented self, the family (group)-oriented self and the other-oriented self], finding that the essential connotations of Chinese people's self-actualization had three fields, i.e., “to become oneself completely,” “repay the family with personal achievements,” and “expending personal well-being to serve the community.” The first of these fields belonged to the individual-oriented self, whereas the second and third fields clearly belonged to the self-actualization of the social-oriented self. Although these results do not fully echo the concept of the four-part Chinese self, a step forward has indeed been taken.

Attempts related to the four-part theory of the Chinese self have been made in developing self-esteem scales; Weng and Yang (2003) attempted to conduct a conceptual analysis and scale development for social-oriented self-esteem and individual-oriented self-esteem in which the social-oriented self-esteem is divided into relationship-oriented self-esteem, familistic (group)-oriented self-esteem, and other-oriented self-esteem, resulting in four types of self-esteem when individual-oriented self-esteem is added. Based on the four-part Chinese self, Weng and Yang (2003) developed pre-test questionnaires on “The multi-part self-esteem scale for Chinese people” to investigate undergraduates from both Taiwan and Mainland China. The data obtained generated six oblique factors: “personal ability and independence,” “physical health and appearance,” “interpersonal relations and popularity,” “emotions and interactions of family members,” “family background and economy,” and “social identity and care.” The first and second factors pertain to individual-oriented self-esteem (especially the first factor), the third factor belongs to relationship-oriented self-esteem, the fourth and fifth factors belong to family-oriented self-esteem, and the sixth factor belongs to other-oriented self-esteem. They integrate six subscales of self-esteem into the formal “Multi-part self-esteem scale of Chinese people” (Weng and Yang, 2003; Yang, 2004).

## This present research

Yang believes that the four-part theory of the Chinese self can completely cover the Chinese self and that, being mutually distinctive, the four types of self represent the methods of interaction by which the Chinese interact with others in the four major modalities. We believe that Yang's four-part theory of the Chinese self is a very important theory, not only having indigenous compatibility and depicting the self-construal of Chinese people deeply and properly but also distinguishing between the Chinese self and the Western self. However, except for the two previous studies authored by Yang (Weng and Yang, 2003; Lu and Yang, 2005), other studies focus exclusively on the differences between the individual-oriented self and the social-oriented self (e.g., Sun and Wang, 2005), failing to distinguish among the three types of social-oriented self—i.e., the relationship-oriented self, the familistic (group)-oriented self, and the other-oriented self.

In this study, we wanted to test (both experimentally and directly) different types of self. In particular, we wanted to distinguish among different types of self under the social-oriented self, to investigate (1) how to effectively trigger different types of self, especially to distinguish the relationship-oriented self, the familistic (group)-oriented and the other-oriented self; and (2) the relative importance of the three types of self—i.e., the relationship-oriented self, the familistic (group)-oriented, and the other-oriented self. “Non-specific others” involved in the other-oriented self are particularly noteworthy. The “non-specific others” proposed by Yang (1995, 2004) are a large number of anonymous and generalized other persons, whereas what is intended by the “other-oriented self” is that the individual's words and deeds satisfy the expectation of these non-specific others, be honored and earn a face in public. So-called “non-specific others” hint at a larger number of people that must observe the same cultural or social norms as the individual. In other words, when we say, “I do not know what others would think,” those “others” should be others who share our cultural or social norms. In this study, we will therefore use this concept as the basis of discussion and our research design.

In addition, the relationship-oriented self proposed by Yang was originally defined as an intimate dyadic relationship such as the relationship between husband and wife, parent and child, etc. In this theory, the primary motive of satisfaction that is expected from the relationship-oriented self is that of mutual support, mutual reception, and sharing. Because we believe this definition is more appropriate when referring to equal-status relationships, we first define the relationship-oriented self as the self in the relationship between spouses. However, according to Yang's theory, relationships such as that between parent and child can also be viewed as involving the relationship-oriented self and shows some overlap with the familistic (group)-oriented self when an attempt is made to differentiate the two. Therefore, our basic idea is to use the distinctive “spouse/lover” relationship for discussing relationship-oriented self. If such a relationship is indistinct from that implicated by the familistic (group)-oriented self, then the four-part concept of Chinese self needs to be modified.

To be more specific, in the four-part theory of the Chinese self, Yang (2004) provides a clearer description on self, arguing the three sub-divisions of the social-oriented self is characteristic of the Chinese self. In this study, three experiments were conducted to examine how to effectively trigger different types of self and the relative importance of the three types of self. In Experiment 1, the photo-primed method would be used to demonstrate that the three types of self can be individually triggered and indeed exist. In Experiment 2, we adopted the research model of the limited self-regulation resource to investigate the importance of different types of self. In Experiment 3, the relative importance of three types of self in the social-oriented self was investigated through different scenarios of conflict between different types of self and through the individuals' choices, the relative importance of different types of self was studied.

## Experiment 1

In examining the four-part theory of the Chinese self, it is most important to confirm the existence of each of the four types of self, especially the relationship-oriented self, the familistic (group)-oriented self and the other-oriented self, which are under the social-oriented self and can be individually triggered. In addition, although we believe that the individual-oriented self and the social-oriented self are distinguishable from one another, we also believe that the three types of social-oriented self are not clear-cut but instead unique, albeit with overlapping. We wish to test these ideas.

In Experiment 1, the photo-primed method was employed. Sun (2007), Cheng (2006), Chen (2013), and Sun and Kao (2017) used photos to prime the individual-oriented self and social-oriented self in individuals. In this study, photos related to different types of self were used to prime various orientations of self. We hypothesized that once the individual-oriented self has been primed, an individual's reaction time for an individual-related word will be significantly shorter than for other categories of words. When the relationship-oriented self is primed, an individual's reaction time for a word related to the relationship orientation of two persons will be significantly shorter than for other categories of words. When the familistic (group)-oriented self is primed, an individual's reaction time for a family (group) orientation-related word is significantly shorter than for other categories of words. When the other-oriented self is primed, an individual's reaction time for other orientation-related words is significantly shorter than for other categories of words. We also predicted that when the relationship-oriented self, the familistic (group)-oriented self, and the other-oriented self (all of which are under the social-oriented self) are primed, individuals will have a significantly shorter reaction time for cross-category interpersonal words, i.e., words that reference the common aspects of basic concepts.

### Methods

#### Participants and design

The participants were 88 college students from a national university in Taiwan participated for course credit. Six participants who had extreme reaction times were excluded. There were five different self-priming conditions, which were individual-oriented self, the relationship-oriented self, the familistic (group)-oriented self, the other-oriented self, and neutral condition. Participants in each group were only shown photos of one type according to their assigned group and asked to respond to words from all six categories. The dependent variable was the participant's reaction time to words from various categories.

#### Materials

The materials included photos that use to prime different types of self and included different categories of words/phrases that can reflect different types of self.

##### Photos triggering different types of self

In this experiment, five types of photos—one-person photos, two-person photos, family photos, social-activities photos, and natural-scenery photos—10 photos per type, were used. Pictures of an individual who is reflecting, at work, or participating in an activity were used to prime the individual-oriented self. Pictures of couples or lovers holding hands, smiling to each other or engaging in activities together were used to prime relationship-oriented self. Pictures of family gatherings, e.g., a family at the Spring Festival dinner table, clan reunion events, etc., were used to prime the familistic (group)-oriented self. Pictures such as those portraying a group of Chinese fans at a China-South Korea baseball game were used to prime the other-oriented self. Common natural landscape pictures were used as the neutral priming control group.

##### Word/phrase categories

In the pre-test, three graduate students who know the four-part theory well-worked together to select words that could reflect the individual-oriented self, the relationship-oriented self, the familistic (group)-oriented self and the other-oriented self. Both words of individual-oriented self and words covering the three types of social-oriented self (cross-category interpersonal words) from the “Frequency Dictionary of Written Chinese” published by the Academia Sinica (Chinese Knowledge Information Processing Group, 1994), were then included in the questionnaire. We then explained to a group of respondents about the meanings of these different selves and asked them to judge both the category to which a word should belong (multiple choices were allowed) and a word's degree of association with the self-category to which it was assigned (the degree was measured on a seven-point scale; the higher the number, the greater the degree of association).

The self-category to which each word belongs was determined based on the respondents' choices. The criteria of word selection were that the word was categorized into a particular self-category by more than 90% of the respondents, that the word's degree of association with the self-category was scored at 5 or above and that the percentages at which the word was assigned to other self-categories were all below 10%, thus confirming that the word was attributed to a particular self-category. The selection criteria of cross-category interpersonal words were both that a word had a percentage of over 50% [at which it was categorized into two-person relationship orientation, family (group) orientation, or other orientation] and that the word had a degree of association of 5 or above with its category.

After these pre-tests, a total of 358 respondents determined words from six self-categories: the individual self-category (e.g., 自主 “autonomy”, 獨特 “uniqueness”), the two-person relationship self-category (e.g., 甜蜜 “sweet”, 卿卿我我 “deeply attached to each other”), the family (group) self-category (e.g., 孝順 “filial piety ”, 長幼有序 “to respect for seniority”), the other-orientation self-category (e.g., 社會認可 “social recognition”, 公德心 “civic-minded”), neutral words (e.g., 濕潤 “moist”, 彎曲 “bend”), and cross-category interpersonal words (e.g., 和睦 “harmony”, 在一起“togetherness”). Each self-category included ten words or phrases.

#### Procedure

After arriving at the laboratory, the participant was informed that the experiment was designed to understand an individual's ability of free association. After filling the consent form, each participant was then randomly assigned to see one of the five types of photos—one-person photos, two-person photos, family photos, social-activities photos, or natural-scenery photos. Photos were presented one at a time, and followed by a word/phrase. Participants need to decide if the word/phrase could reflect the thought elicited by the photo as fast as they can by pressing the “yes” or “no” key, and the reaction times were all recoded. Photos from each of the five types would paired with words/phrases from all the self-categories and neutral word category. All experimental materials were presented *via* computers *using MediaLab software*.

### Results

After applying an inverse transformation on reaction time data, the assessments of the normality and sphericity assumptions were acceptable (Box and Cox, 1964). For ease of interpretation, raw reaction times will be displayed for descriptive purposes only. A two-way mixed design analysis of variance (ANOVA) with a between-subjects factor of five different self-priming conditions and a within-subjects factor of six word categories revealed a significant interaction between self-priming and word categories, *F*(20, 415) = 9.548, *p* < 0.001, $\eta_{p}^{2}$ = 0.315. To test the hypotheses, separate repeated measures one-way ANOVAs were conducted in different self-priming conditions.

In the individual-oriented self-priming condition, a repeated measures one-way ANOVA revealed a statistically significant effect on word categories, *F*(5, 85) = 12.305, *p* < 0.001, $\eta_{p}^{2}$ = 0.420. *Post-hoc* comparisons were then performed. The participants had a significantly faster average reaction time to the individual-orientation self-category words (*M* = 845.03) than to the two-person relationship-orientation self-category words (*M* = 919.03, *p* = 0.042), the family (group)-orientation self-category words *(M* = 994.26, *p* < 0.001), the other-orientation category words (*M* = 1117.61, *p* < 0.001), the neutral words (*M* = 1015.31, *p* < 0.001), and the cross-category interpersonal words (*M* = 952.98, *p* = 0.003). This result was consistent with expectations.

In the relationship-oriented self-priming condition, a repeated measures one-way ANOVA was conducted and result showed a statistically significant effect on word categories, *F*(5, 75) = 4.964, *p* < 0.001, $\eta_{p}^{2}$ = 0.249. *Post-hoc* comparisons indicated that participants who were primed relationship-oriented self had a significantly faster average reaction time to the two-person relationship-orientation self-category words (*M* = 1005.93) than to the individual-orientation self-category words (*M* = 1338.85, *p* = 0.001), the family (group)-orientation self-category words (*M* = 1312.48, *p* < 0.001), the other-orientation self-category words (*M* = 1273.51, *p* < 0.001), and the neutral words (*M* = 1216.17, *p* = 0.003), whereas there was no significant difference related to the cross-category interpersonal words (*M* = 1085.53, *p* = 0.065).

In the familistic (group)-oriented self-priming condition, a repeated measures one-way ANOVA revealed a statistically significant effect on word categories, *F*(5, 80) = 12.978, *p* < 0.001, $\eta_{p}^{2}$ = 0.448. *Post-hoc* comparisons showed that the participants had a significantly faster average reaction time to the family (group) orientation self-category words (*M* = 1032.65) than to the individual-orientation self-category words (*M* = 1241.41, *p* = 0.006), the two-person relationship-orientation self-category words (*M* = 1519.93, *p* < 0.001), the other-orientation self-category words (*M* = 1540.85, *p* < 0.001), and the neutral words (*M* = 1214.04, *p* = 0.004), whereas there was no significant difference related to the cross-category interpersonal words (*M* = 1131.50, *p* = 0.150).

In the other-oriented self-priming condition, a repeated measures one-way ANOVA also revealed a statistically significant effect on word categories, *F*(5, 85) = 2.981, *p* = 0.016, $\eta_{p}^{2}$ = 0.149. *Post-hoc* comparisons showed participants who were triggered the other-oriented self had a significantly faster average reaction time to the other-orientation self-category words (*M* = 1038.96) than to the individual-orientation self-category words (*M* = 1299.79, *p* = 0.005), the family (group)-orientation self-category words (*M* = 1268.91, *p* = 0.009), and the neutral words (*M* = 1227.48, *p* = 0.039), whereas there was no significant difference related to the two-person relationship-orientation self-category words (*M* = 1098.79, *p* = 0.390) or the cross-category interpersonal words (*M* = 1124.82, *p* =.076). The results are presented in Table 2.

**Table 2:** *Post-hoc* comparisons of reaction time among different groups (raw data).

	***M(SD)***		***M(SD)***	***SE***	***p***
**TRIGGERING THE INDIVIDUAL-ORIENTED SELF (*****N*** = **18)**					
Individual-orientation self-category words	845.03 (163.8)	Two-person relationship-orientation self-category words	919.03^*^ (204.62)	31.78	0.042
		Family (group)- orientation self-category words	994.26^**^ (230.67)	37.86	0.000
		Other- orientation self-category words	1117.61^**^ (327.86)	61.42	0.000
		Neutral words	1015.31^**^ (219.25)	32.52	0.000
		Cross-category interpersonal words	952.98^**^ (225.50)	34.33	0.003
**TRIGGERING THE TWO-PERSON RELATIONSHIP-ORIENTED SELF (*****N*** = **16)**					
Two-person relationship-orientation self-category words	1005.93(284.79)	Individual-orientation self-category words	1338.85^**^ (540.04)	110.01	0.001
		Family (group)- orientation self-category words	1312.48^**^ (380.58)	76.62	0.000
		Other- orientation self-category words	1273.51^**^ (357.96)	52.95	0.000
		Neutral words	1216.17^**^ (281.62)	62.33	0.003
		Cross-category interpersonal words	1085.53 (250.79)	58.03	0.065
**TRIGGERING THE FAMILISTIC (GROUP)-ORIENTED SELF (*****N*** = **17)**					
Family (group)- orientation self-category words	1032.65(302.04)	Individual-orientation self-category words	1241.41^**^ (421.66)	57.88	0.006
		Two-person relationship-orientation self-category words	1519.93^**^ (465.71)	77.15	0.000
		Other- orientation self-category words	1540.85^**^ (439.37)	83.91	0.000
		Neutral words	1214.04^**^ (324.84)	49.19	0.004
		Cross-category interpersonal words	1131.50 (429.30)	57.00	0.150
**TRIGGERING THE OTHER-ORIENTED SELF (*****N*** = **18)**					
Other- orientation self-category words	1038.96(283.47)	Individual-orientation self-category words	1299.79^**^ (459.17)	98.23	0.005
		Two-person relationship-orientation self-category words	1098.79 (297.95)	68.14	0.320
		Family (group)- orientation self-category words	1268.91^**^ (457.69)	95.57	0.009
		Neutral words	1227.48^*^ (424.87)	74.07	0.039
		Cross-category interpersonal words	1124.82 (273.50)	67.54	0.076
**TRIGGERING THE NEUTRAL CONDITION (*****N*** = **19)**					
Neutral words	975.64(224.03)	Individual-orientation self-category words	1050.59 (318.85)	64.52	0.402
		Two-person relationship-orientation self-category words	982.62 (193.46)	44.34	0.766
		Family (group)- orientation self-category words	1049.64 (352.17)	70.50	0.632
		Other- orientation self-category words	1023.47 (261.90)	61.98	0.300
		Cross-category interpersonal words	1049.16 (329.98)	71.71	0.461

*All times in are in milliseconds. Asterisks indicate a significant effect*.

* *p < 0.05*,

** *p < 0.01*.

### Discussion

The results showed that when the individual-oriented self was primed, the participants had a significantly faster reaction time to the individual-related words than to the individual-unrelated words; as expected, the three types of social-oriented self were distinguished from each other while sharing an underlying basis. Therefore, when the relationship-oriented self was primed, the participants had a significantly faster reaction time to the words reflected the relationship-oriented self than to words of other self-categories, along with an accelerated reaction time to the cross-category interpersonal words, whereas they exhibited no significant difference between the reaction time to the two-person relationship orientation self-category words.

When the familistic (group)-oriented self was primed, the participants had a significantly faster reaction time to words reflected the familistic (group)-oriented self than to words of other categories, along with an accelerated reaction time to the cross-category interpersonal words; they exhibited no significant difference between the reaction time to the cross-category interpersonal words and the reaction time to words from the familistic (group)-oriented self-category. When the other-oriented self was primed, the participants had a significantly faster reaction time to words reflected the other-oriented self than to words related to the familistic (group)-oriented self; they exhibited a significantly accelerated reaction time to cross-category interpersonal words, whereas they exhibited no significant difference in reaction time involving words related to the other-oriented self. These results essentially supported the four-part theory of the Chinese self proposed by Yang that in addition to the individual-oriented self, the social-oriented self can be further divided into three different types of self that are distinctive but do not necessarily exist independently.

## Experiment 2

In Experiment 2, we wanted to investigate the importance of different types of self in the social-oriented self and adopted the research model of the limited self-regulation resource. Muraven and Baumeister (2000) argued that so-called holistic self-regulation is a finite resource and can be temporarily depleted when it is used. Each self-control task performed by an individual is bound to consume resources, and the more those resources are used, the greater the task's effect on the performance of the sequent task (Baumeister et al., 1998; Muraven et al., 1998). Muraven et al. (1998) asked participants to watch a clip of uncomfortable film and asked them to maximally exaggerate, suppress or naturally express their emotions and facial expressions while watching the film clip. The participants were then asked to perform a hand-squeezing task. The results showed that the participants who exaggerated or suppressed their emotions and facial expressions performed more poorly in the squeezing task than those from the control group, who expressed their emotions naturally.

Muraven and Baumeister (2000) argued that the reason for their result is that the suppression or exaggeration task requires self-control and thus consumes self-regulation resources: after the participants perform the task, they will be affected when performing other tasks that require resource-consuming self-control. Crocker et al. (2006) argue that when individuals find something that is either important to them or relevant to their self-worth, they will invest more self-control resources in the relevant task; the more difficult the task, the more resources they will consume and the worse they will perform in the subsequent task. For example, the more relevance attributed to learning for an individual's self-worth and the more difficult a learning task, the worse individuals will perform in a subsequent task. In Experiment 2, the same logic was adopted to test the importance of different orientations of self.

The purpose of Experiment 2 was to examine the importance of three types of social-oriented self. Participants were asked to refute after reading a short essay on the importance of a particular type of self. According to the self-regulation concept, rebuttal of the importance of different types of self is very difficult and thus consumes resources; the more importance attributed to a type of self by the individuals, the more resources they will consume in performing the rebuttal and the worse they will perform in the subsequent task. Therefore, the participants' performance in the subsequent task can be used to test the importance of different types of self.

### Method

#### Participants and design

The participants of this study were college students from a national university in northern Taiwan. The valid sample size was 55 persons after excluding 4 outliners in the number-comparing tasks; 43 participants were female and 12 were male. All participants were 19 to 22 years of age (*M* = 20.02). This experiment was a one-factor between-subject design in which participants were randomly assigned to four scenarios to rebut the importance of different types of self (independent variables), i.e., the relationship-oriented self, the familistic (group)-oriented self, the other-oriented self, and the “convenience store” group (the control group).

#### Materials

##### Essays related to different types of self

After the pre-test, one essay was selected for each of the three types of self; one essay on the importance of convenience stores was used as the control group. These essays showed no significant differences in rationality and persuasiveness. The participants were asked to write a ten-argument rebuttal in 10 min after reading the essay, each of which started, “I do not agree with that view, because …” For example, the participants assigned to refute the relationship-oriented self were given the following short essay:

“*I believe that to maintain a good relationship between lovers (or husband and wife), both mind and effort are required; when two are getting along, in addition to enjoying the sweetness of life, they should be considerate and take care of each other. When making a major decision, the two (lovers or couple) should trust each other and have conversations. Because another half is a part of oneself and very important spiritual support, in a relationship, one should accept the other's imperfection, try to be proud of the other and strive to contribute to making each other happy.”*

Other participants assigned to refute the familistic (group)-oriented self or other-oriented self received different essays and were asked to formulate a rebuttal after reading the essays. Participants in the control group were assigned to read an essay on the importance of convenience stores to our lives and to formulate a rebuttal.

##### The measurements of manipulations

To confirm that there were no significant differences among the participants with respect to their level of comprehension of, level of agreement with and importance assessment of the descriptive essay about different types of self, the participants were asked to make an assessment using a seven-point scale. The higher the score, the higher the level it represents.

To confirm that the participants did their best on the rebuttal tasks, the participants were also asked to perform self-evaluations on their perceived effort and the persuasiveness of their completed tasks, on a seven-point scale. The higher the score, the higher the level it represents.

##### Self-esteem scale

To prevent the participant's self-esteem level from affecting his/her performance in completing the rebuttal task, the participant's self-esteem was measured as the control variable using the “Self-Esteem Scale” (Rosenberg, 1965), which had 10 items and was measured by a seven-point scale. The higher the score, the higher the level of self-esteem.

##### Number-comparing task

To understand the remaining resources of participants from different groups after performing the rebuttal task related to the self of different orientations, the participants were asked to perform a number-comparing task. Each question involved two numbers with an identical number of digits (4–12 digits); if the participant thought that the two numbers were identical (e.g., “553314521705” and “553314521705”), “O” was marked, whereas if the participant thought that the two numbers were different from each other (e.g., “537608031077” and “537806031077”), “X” was marked.

#### Procedure and measures

The experimenter informed all of the participants that the experiment purported to collect college students' opinions. After filling the consent form, each participant was then randomly assigned an essay and asked to read it. First, each participant was assessed on his/her comprehension of the essay, the extent of his/her agreement with its content and the importance of the content. Next, each participant was informed that there were enough opinions supporting the content of the essay and asked to do his/her best to think of and write down (in the space of 10 min) 10 counterarguments. After a participant completed the rebuttal task, he/she was asked to perform a self-evaluation of his/her perceived effort in completing the rebuttal task and the persuasiveness of his/her counterarguments.

After a participant completed the self-evaluation, he/she was informed that before proceeding to the next step, he/she was required to perform a number-comparing task to monitor his/her attention and collect data for another experiment. Each participant was informed that he/she should try his/her best, that there was no limitation on the number of questions and that he/she could stop at any time. The number of number-comparing questions completed by the participant was set as the dependent variable. A smaller number represents the consumption of more self-resources in performing the rebuttal task.

After each participant completed this task, he/she then was informed that the experiment was over. Finally, explanations about the rationale of the study were given and gratitude was expressed to all participants for their participation.

### Results

#### Manipulation checks

##### Comprehension and importance of the essay

The assessments on a participant's comprehension, agreement and importance on the essay were used as the dependent variables, and multivariate analysis of variance (MANOVA) was conducted on “rebuttal on scenarios of different types of self” to understand whether there were differences in the assessments on the three items among four groups of participants. The results showed that the overall effect was insignificant (Wilks' Λ = 0.64, *ns*), indicating that the four groups of participants showed no differences in the assessments of their comprehension, agreement and importance evaluation related to their essays. In addition, the average score of each group on each of the indicators was above 5.7, indicating that the participants had a high level of comprehension of and agreement on the essay, while regarding its content as very important. Therefore, the manipulation of the essays was successful.

##### The perceived effort in performing the rebuttal task

The participants' self-evaluations of their effort on the rebuttal task and the persuasiveness of their counterarguments were set as the dependent variables, and a MANOVA was conducted to determine whether there were differences in the two items among four groups of participants. The results showed that the overall effect was insignificant (Wilks' Λ = 0.92, *ns*), indicating that the four groups of participants showed no difference in their self-assessed effort in completing the rebuttal task, with an average score of 5.89 points. No significant difference was found in the persuasiveness of counterarguments, with an average score of 4.82 points, indicating that each group performed the rebuttal task in accordance with the instructions and that the manipulation of the rebuttal task was successful.

#### The number of number-comparing questions completed

The number of number-comparing questions completed by the participant was set as the dependent variable, the “rebuttals on scenarios of self of different orientations” was set as the independent variables, and the individual's self-esteem score was set as the control variable for conducting the single-factor analysis of covariance (ANCOVA). The results showed that the effect of refuting different orientations of self was significant [*F*(3, 50) = 4.32, *p* = 0.009, $\eta_{p}^{2}$ = 0.206], indicating that different rebuttal scenarios consumed different levels of self-regulation resources.

**Figure 1:**
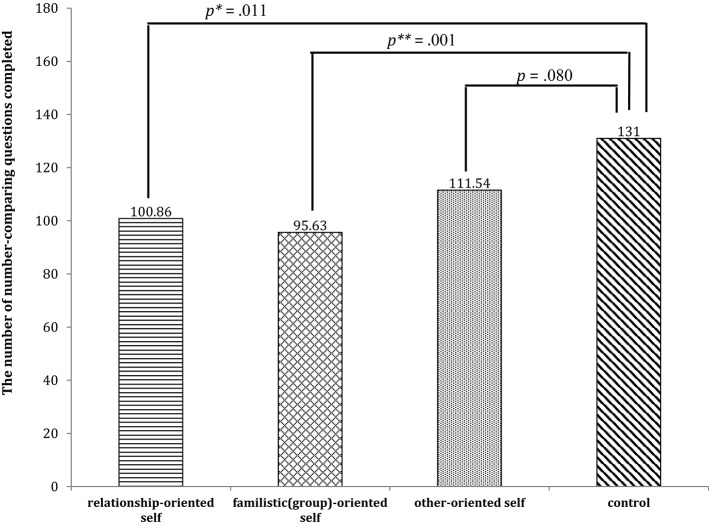
The average number of number-comparing questions completed by different groups.

*Post*-*hoc* comparisons were performed using LSD (least significant difference) tests. The results showed that participants from the three groups whose rebuttals related to three orientations of self completed fewer of the number-comparing questions. This was particularly true of the group of participants who refuted the relationship-oriented self and the group of participants who refuted the familistic (group)-oriented self, both of which had results significantly different from the control group (*p* = 0.011; *p* = 0.001). Conversely, the group that refuted the other-oriented self showed a marginally significant difference from the control group (*p* = 0.080). These results indicate that for the participants, regardless of which type of self they were refuting, the refuting process consumed a remarkable amount of their self-regulation resources, making it difficult for them to concentrate on completing the subsequent task. This was especially true of participants who refuted the relationship-oriented self and family-oriented self. Participants who refuted the other-oriented self also showed a similar tendency. The results are presented in Figure 1.

### Discussion

In Experiment 2, we attempted to use the concept of limited overall self-regulation resources to test the importance of different types of self. Based on the idea that participants would write counterarguments after reading essays on different types of self and that the particular type of self implicated was important for a particular participant, rebuttal of a particular self orientation would cost more resources and led to poor performance in the subsequent task. It was found that the relationship-oriented self, the familistic (group)-oriented self, and the other-oriented self (all of which are under the social-oriented self) were all important self orientations, causing the participants' poor performance in the subsequent task, especially for participants who refuted the relationship-oriented self and family-oriented self. Participants who refuted the other-oriented self showed a similar tendency. However, these results did not reveal the relative importance of different types of self, an issue that was addressed in Experiment 3.

## Experiment 3

In Experiment 2, the importance to each individual of the three orientations of self was demonstrated. The question that remains is this: what is the relative importance of the three different types of self to each individual? In other words, when conflict between two orientations of the self occurs in a particular situation and context, which orientation of the self is more important to the individual? This issue has yet to be clarified.

In Experiment 3, the relative importance of various types of social-oriented self was investigated through different scenarios of conflict between different types of self. For example, Chinese revolutions have espoused the notion of “exchanging the small-love for the big-love” in which small-love refers to the love between couples, representing the relationship-oriented self, whereas big-love refers to care for a great many others, representing the other-oriented self. “Exchanging the small-love for the big-love” represents a conflict between the relationship-oriented self and the other-oriented self. In this experiment, situations of conflict between various orientations of self were set up. Through the participants' choices, the relative importance of different types of self was analyzed.

In this study, we hypothesized that when individuals are confronted by two conflicting orientation origins of self, they are bound to choose the type of self that has a more important outcome to them and relinquish the type of self that is relatively unimportant. Therefore, we performed pairwise comparison of the three orientations of self, i.e., three groups of pairwise comparison. For each group, four scenarios were designed and the participants were asked to make a choice in each conflict scenario. Ultimately, by analyzing the participants' choices, the relative importance of different types of self was understood.

### Method

#### Participants and design

The participants in Experiment 3 were 63 college students from public universities in northern Taiwan, including 48 females and 15 males. Their average age was 19.10 years. Participants were randomly assigned to scenarios of conflict between any two of the three orientations of self: (1) the relationship-oriented self vs. the familistic (group)-oriented self; (2) the relationship-oriented self vs. the other-oriented self; and (3) the familistic (group)-oriented self vs. the other-oriented self.

#### Materials

##### Scenarios of conflict between different orientations of self

Experiment 3 was designed to present a participant with a conflict situation that simultaneously triggers selves of different orientations and to ask the participant to make a choice in the dilemma. The goal is to understand the relative importance of the three types of social-oriented self. Therefore, three types of conflict scenarios were needed. To further evaluate the possibility that requirements from any two orientations of self in the three conflict scenarios are the same (i.e., behaviors related to the self of a certain orientation are not more reasonable or more likely to occur than behaviors related to the self of another orientation), in the pre-test, each scenario that contains different conflicting orientations of self was individually divided into two versions, A or B, in which behaviors related to only one orientation of self occur. Next, the two versions that contain behaviors related to a single orientation of self were compared to determine whether they demonstrate the same possibilities.

The pre-test was a one-way between-subject design with 21 groups of dilemmas. Two versions (A and B) for each dilemma situation were created, each of which contained a single type of orientation of self. The participants were randomly assigned to read either the A or the B version. For example, the “dilemma of family (group) self vs. the other-oriented self” is as follows:

“*Meixiu, who is in critical condition, intends to sign an organ-donation agreement (consistent with a doctor's recommendation) to help many patients in need. However, when her closest family members hear about her intention, they feel very sad and think she should leave her body intact. Therefore, they are opposed to her plan to donate organs.*”

In the pre-test, this dilemma is made into the A version of “the other-oriented self”:

“*Meixiu, who is in critical condition, intends to sign an organ-donation agreement (consistent with a doctor's recommendation) to help many patients in need.*”

The B version of a “request from the familistic (group)-oriented self” is as follows:

“*Meixiu is critically ill. Her close family members are quite sad and they hope to preserve the physical integrity of her body. They are opposed to donating her organs*.”

Eighty six college students from universities in northern Taiwan were randomized assigned to received either the A version or B version of the questionnaire and assessed the probability that they would agree to the choice set out for the situation using a seven-point scale (the higher the score, the more likely the participant is to agree to act the same way).

The probability that each participant believes he/she would engage a certain behavior was then set as the dependent variable and “the behavior in a situation with a single orientation of the self” was set as the independent variable to perform the two-tailed *t*-test. Ultimately, among the three conflicting situations, two orientations of self that showed no significantly different probabilities of behaviors and that had a probability of above 4.5 points in each case were chosen. Four scenarios of each type of conflict were designed, resulting in 12 scenarios overall.

Finally, based on the pre-test results, 12 scenarios of conflict in three categories were chosen:

The first category involved scenarios featuring conflicts between the relationship-oriented self and the familistic (group)-oriented self. For example, in this scenario, the husband experiences a dilemma when he wants to take care of his wife and pursue rights for the couple while pursuing the interests of his family group. In other words, this is a conflict scenario of the relationship-oriented self and the familistic (group)-oriented self.

The second category involved scenarios featuring conflicts between the relationship-oriented self and the other-oriented self. For example, the actor experiences a dilemma in which he must care for either the relationship-oriented self or the other-oriented self in a situation involving concerns about other's thoughts and social honor.

The third category involves scenarios featuring conflicts between the familistic (group)-oriented self and the other-oriented self in which the actor experiences a dilemma involving the need to take care of either the interests of the family group to which he belongs or the other-oriented self. Such a scenario involves concerns about other's thoughts and social honor.

#### Procedure and measures

After arriving at the laboratory, the participant was informed that the experiment was designed to understand an individual's choices related to specific life situations. The experimenter might also emphasize that these situations involved actual events that befell the participant's classmates and alumni, thus emphasizing the real-life nature of the situations so that the participant would be more engaged in the situation. The experimenter then asked the participant to imagine that he/she is the person in the situation experiencing the dilemma. The participant then read the descriptions of all of the scenarios and indicated which choice he/she would make if he/she were the person in the situation. The main dependent variables were the participant's choices in the dilemma. The participants also needed to measure their levels of certainty about their choices using a seven-point scale. The higher the score is, the more certain the participant's choice. After the participant completed all of the questionnaires, the experiment was completed. The experimenter then debriefed and thanked the participant for his/her participation.

### Results

#### Selection of different types of self

The participants' choices were used as dependent variables to be tested on fitness. The selected choices and chi-square of each scenario are shown in Table 3. The results showed that in the conflict situation involving “the relationship-oriented self vs. the familistic (group)-oriented self,” the participants emphasized the familistic (group)-oriented self. In the conflict situation involving “the relationship-oriented self vs. the other-oriented self,” the participants were more inclined to choose behaviors facilitating the other-oriented self over the relationship-oriented self; however, there was a scenario about “whether to invite singers to have a show in his/her spouse's company or to perform for the public welfare” that was discarded in the final analysis because many participants were confused because the scenario contained poor definitions. In the conflict situation involving “the familistic (group)-oriented self vs. the other-oriented self,” except for the “bread donation” scenario, in which the participants' choices did not differ significantly, the participants strongly preferred the choices favoring other-oriented self behaviors in the other three scenarios, indicating that in the conflict of “the familistic (group)-oriented self vs. the other-oriented self,” the participants preferred the other-oriented self.

**Table 3:** Choices made by individuals about behaviors involving different scenarios of conflicting orientations of self.

**RELATIONSHIP-ORIENTED SELF VS. FAMILISTIC (GROUP)-ORIENTED SELF**												
**Scenario**	**Financial support to parents**			**Boyfriend/family member tourism**			**Location of rented house**			**Marital considerations**		
	**Relationship**		**Familistic**	**Relationship**		**Familistic**	**Relationship**		**Familistic**	**Relationship**		**Familistic**
Choice	14		48	11		52	20		43	6		57
Percentage	22.2		6.2	17.5		82.5	31.7		68.3	9.5		90.5
χ^2^	18.65			26.68			8.40			41.29		
*p*	0.000			0.000			0.004			0.000		
**RELATIONSHIP-ORIENTED SELF VS. OTHER-ORIENTED SELF**												
**Scenario**	**Help to promote husband's career**			**Donation for public interests**			**Volunteering in a hospital**					
	**Relationship**		**Other**	**Relationship**		**Other**	**Relationship**		**Other**			
Choice	33		30	23		40	22		41			
Percentage	52.4		47.6	36.5		63.5	34.9		65.1			
χ^2^	0.14			4.59			5.73					
*p*	0.705			0.032			0.017					
**FAMILISTIC (GROUP)-ORIENTED SELF VS. OTHER-ORIENTED SELF**												
**Scenario**	**Use of underground water**			**Site for grandpa's birthday celebration party**			**Organ donation**			**Bread donation**		
	**Familistic**		**Other**	**Familistic**		**Other**	**Familistic**		**Other**	**Familistic**		**Other**
Choice	8		55	3		60	4		59	27		36
Percentage	12.7		87.3	4.8		95.2	6.3		93.7	42.9		57.1
χ^2^	35.06			51.57			48.02			1.29		
*p*	0.000			0.000			0.000			0.257		

Next, fitness was tested by combining answers to the scenarios featuring the three types of dilemma situations. The results showed that in the conflict involving “the relationship-oriented self vs. the familistic (group)-oriented self,” the participants were significantly inclined to choose behaviors in accordance with the familistic (group)-oriented self [χ^2^(1, *N* = 311) = 88.45, *p* < 0.001]. In the conflict involving “the relationship-oriented self vs. the other-oriented self,” the participants were significantly inclined to choose behaviors in accordance with the other-oriented self [χ^2^(1, *N* = 189) = 5.76, *p* = 0.02]. In the conflict involving “the other-oriented self vs. the familistic (group)-oriented self,” the participants were significantly inclined to choose behaviors in accordance with the other-oriented self [χ^2^(1, *N* = 252) = 112.00, *p* < 0.001].

#### The level of certainty of the choices

The levels of certainty of the choices made by the participants were combined to serve as the dependent variable to perform the two-tailed *t*-test. The results indicated that in the conflict of “the relationship-oriented self vs. the familistic (group)-oriented self,” the participants' level of certainty about facilitating the familistic (group)-oriented self (*M* = 5.50, *SD* = 1.05) was higher than the participants' level of certainty about facilitating the relationship-oriented self (*M* = 5.12, *SD* = 1.09) [*t*(249) = −2.27, *p* = 0.02]. In the conflict of “the relationship-oriented self vs. the other-oriented self,” the participants' level of certainty of participants about facilitating the other-oriented self (*M* = 5.67, *SD* = 0.97) was higher than the participants' level of certainty about facilitating the relationship-oriented self (*M* = 5.35, *SD* = 0.96) [*t*(249) = −2.25, *p* = 0.03]. In the conflict of “the other-oriented self vs. the familistic (group)-oriented self,” the participants' level of certainty about facilitating the other-oriented self (*M* = 5.81, *SD* = 0.92) was higher than the participants' level of certainty about facilitating the family (group)-oriented self (*M* = 4.98, *SD* = 1.44) [*t*(249) = −4.82, *p* < 0.001].

### Discussion

The results of Experiment 3 showed the participants' level of emphasis on the three types of social-oriented self. In descending order, they were the other-oriented self, the familistic (group)-oriented self, and the relationship-oriented self. This tendency was exhibited not only in the choice preference in the conflict situations but also in the participants' level of certainty about their choices, indicating that when confronted by a dilemma, individuals seemed to have a tendency to “pursue the interests of the overall situation” (顧全大局). They tended to safeguard the familistic (group)-oriented self when experiencing a conflict between the relationship-oriented self and the familistic (group)-oriented self, whereas in the conflict between the other-oriented self and the familistic (group)-oriented self, individuals demonstrated a remarkable tendency to safeguard the other-oriented self. The results appear to confirm the tendency toward “sacrificing the small-self for the big-self” that is often mentioned in Chinese culture.

## General discussion

Numerous studies have discussed the self of individuals, most frequently mentioning the division between the independent self and the dependent self (Markus and Kitayama, 1991, 2010). Yang (1993, 2004) proposed the four-part theory of the Chinese self for individuals influenced by Chinese culture, in which in addition to the individual-oriented self, the social-oriented self is subdivided into three different types: the relationship-oriented self, the familistic (group)-oriented self and the other-oriented self. Yang believes that this multi-faceted description of self can completely cover the Chinese self. Although his theory does have deep indigenous compatibility and has attracted a great deal of attention, there remains a lack of in-depth empirical investigation. This study was intended to address that inadequacy and attempted to understand whether the three types of social-oriented self are indeed distinctive to individuals and their relative importance to each other.

### Although three types of social-oriented self share a common basis, they are distinctive

In Experiment 1, the self of different orientations was primed through photos. The results showed that as expected, selves of different orientations could be primed individually. For example, when the familistic (group)-oriented self was primed, the participants had a significantly shorter reaction time for words related to the familistic (group)-oriented self than for words reflected the relationship-oriented self or the other-oriented self. A similar phenomenon appeared when the relationship-oriented self or the other-oriented self was triggered, showing that the three types of self can be individually triggered. Because the three types of self all belong to the social-oriented self, they must have a common basis, as also confirmed by the experimental results. These results suggest that regardless of which of the three types of self was triggered, the participants' response time to cross-category interpersonal words significantly accelerated, supporting the hypothesis of a “common basis.” This basis should combine the tendency toward high homonomy (with one's surroundings) and low personal autonomy emphasized by Yang, which prompts Chinese individuals to attach importance to upholding harmonious interpersonal relationships and proper behavior. The characteristics, connotations, and operating principles in different modalities determine specific interaction objects and modes, ultimately positioning and shaping the self of different orientations.

### Each of the three types of self is important

In Experiment 2, the importance of each of the three types of social-oriented self [the relationship-oriented self, the familistic (group)-oriented self, and the other-oriented self] to each individual was investigated using the paradigm of limited self-regulation resources. After reading essays related to different types of self, the participants were asked to write counterarguments. In general, when a certain type of self is important to an individual, that individual consumes more resources to write the rebuttal, leading to poor performance in completing the next task. In the experiment, the participants in the control group were asked to write a rebuttal to an essay claiming that convenience stores make life easier. Generally, the participants in the control group must consume substantial self-regulation resources in writing the rebuttal because in Taiwan, convenience stores are ubiquitous and facilitate daily life, allowing people to buy food and grocery, pay bills, withdraw cash, shop, and send packages. Therefore, it is very difficult to refute the notion that convenience stores make life easier.

Besides, to eliminate the possibility that the results might be attributed to the difference in the ease of essay to counter-argue with, we pretested these essays with caution to ensure there were no significant differences in their persuasiveness and rationality. Furthermore, in experiment 2, the manipulation check also indicated that participants comprehended and agree with the content of these essays equally well. Therefore, the results of the experiment showed that rebuttals of arguments about the self of different orientations were more resource-intensive than rebuttal of the essay about convenience stores, indicating that the three types of social-oriented self were very important to individuals, thus providing further support for the four-part theory of the Chinese self.

### The importance of the other-oriented self is the most undeniable

Although the three types of social-oriented self are all important to varying degrees, in reality, there are situations in which two types of self conflicts, raising the following question: what is the relative importance of each type of self? In Experiment 3, pair-wise conflict of two different orientations of self was designed to attempt to trigger two types of self simultaneously so that an individual can choose between the two and the relative importance of each type of self can be tested. The results showed that when the relationship-oriented self conflicts with the familistic (group)-oriented self, individuals clearly prefer the familistic (group)-oriented self. When the relationship-oriented self conflicts with the other-oriented self, individuals significantly lean toward the other-oriented self. When the familistic (group)-oriented self conflicts with the other-oriented self, individuals significantly choose the other-oriented self.

Overall, the choices made by an individual experiencing conflicts between different types of self reveal the levels of importance that he/she attaches to each of the three types of social-oriented self, which is, in descending order: the other-oriented self, the familistic (group)-oriented self, and the relationship-oriented self. This trend is exhibited not only in individuals' choice preference in conflicting scenarios but also in the certainty of those individuals' choices. When experiencing a conflict between the relationship-oriented self and the familistic (group)-oriented self, individuals exhibited a significantly higher level of certainty when choosing behaviors facilitating the familistic (group)-oriented self. When experiencing a conflict between the relationship-oriented self and the other-oriented self, individuals exhibited a significantly higher level of certainty when choosing behaviors facilitating the other-oriented self. When experiencing a conflict between the other-oriented self and the familistic (group)-oriented self, individuals exhibited a significantly higher level of certainty when choosing behaviors facilitating the other-oriented self.

These results seem to demonstrate that when confronted by such dilemmas, Chinese individuals exhibit a tendency to “pursue the interests of the big-self.” According to the four-part theory of the Chinese self, the relationship-oriented self is exhibited in the important dyadic relationship; its interaction objects are often intimate others (e.g., spouses), emphasizing interdependence and sharing. The familistic (group)-oriented self appears in the context of family or an important group. Yang believes that the basic structure and functional unit of the traditional Chinese society is the family. Therefore, Chinese people regard the family as the most important aspect, creating a familism that emphasizes the family and the pursuit of familial unity, honor and harmony. This could also be why individuals attach more importance to the expression of the familistic (group)-oriented self than to the expression of the relationship-oriented self.

The results also showed that when other types of self conflict with the other-oriented self, individuals tended to choose behaviors facilitating the other-oriented self. Why do Chinese individuals care so much about the other-oriented self? According to Yang's four-part theory of the Chinese self, interaction objects involved in the other-oriented self are non-specific others, which can be either real or imaginary persons, e.g., “people” in the expression of “people all look down on me.” The other-oriented self displays correctly in such a context. Chinese want to be impressed and accepted by “non-specific others,” and caring for others' opinions and norms and attending to one's reputation are important revelations of the other-oriented self.

In discussing the concept of “face” within the Chinese society, Hwang (2012) argues that Chinese people are unwilling to “lose face” in front of others—e.g., to violate public morality—because to do so would violate the Confucian self-cultivation requirements related to the individual. The other-oriented self wishes to gain others' respect and the public's recognition via such upbringing or behavior display. Yang (1991) argues that Chinese people regard themselves on the levels of the public self and the private self. The private self is the self-achieved by an individual through keeping secrets from others, whereas the public self is the self-achieved by an individual though “playing” for others. The private self is more stable, whereas the public self is prone to being influenced by or adjusted to others. The public self that “has cardinal principles in mind (識大體) ” appears to be the embodiment of the other-oriented self.

Overall, this study's greatest contribution is its strong support of the four-part theory of the Chinese self. This theory of self related to Chinese people is not just conceptual argument: it is now empirically confirmed. The study is also conducive to the deeper understanding of scholars who are interested in knowing more about the Chinese people. In addition to the individual-oriented self, Chinese people have the socially oriented selves of the relationship-oriented self, the familistic (group)-oriented self, and the other-oriented self. The three types of social-oriented self are mutually distinctive, can be individually triggered and are all very important to the Chinese. In addition, in this study we provided an effective photo-priming procedure to activate different types of social self, future research may considerate to trigger these selves and then investigate further how individuals with different selves would act or perceive in the same and different social contexts.

Finally, we wonder whether this study is limited: do the results of Experiment 3 merely reflect social expectations? Perhaps we cannot completely rule out the possibility, but we did attempt to minimize it. In the pre-test, we assessed the possibility of the presence of each type of self's behaviors and asked the participants to simultaneously evaluate the probability that they and others would behave in a particular way. Later, we constructed scenarios based on the resulting behaviors of two differently oriented selves. Those behaviors are highly possible and are not significantly different from each other in terms of their likelihood. Therefore, there should not be an issue about behaviors of one type of self being more in line with social expectations than those of another type of self. Therefore, the choice made by a participant in Experiment 3 should be his or her decision after careful consideration and can reflect which type of self is more important to the participant.

## Ethics statement

This study was carried out in accordance with the recommendations of the guidelines of Research Ethics, the IRB of National Chengchi University, with written informed consent from all subjects. All subjects gave written informed consent in accordance with the Declaration of Helsinki. The protocol was approved by the IRB of the National Chengchi University.

## Author contributions

The author confirms being the sole contributor of this work and approved it for publication.

### Conflict of interest statement

The author declares that the research was conducted in the absence of any commercial or financial relationships that could be construed as a potential conflict of interest.
